# Perceived sleep disturbance in healthy women with a history of childhood emotional abuse is not reflected in objective sleep measures or nocturnal heart rate variability

**DOI:** 10.3389/fpsyt.2026.1853814

**Published:** 2026-08-05

**Authors:** Samuel Trumm, An Bin Cho, Tina Bidiak, Stefanie Luippold, Christian Otte, Eugenia Kulakova, Katja Wingenfeld

**Affiliations:** 1 Department of Psychiatry and Neurosciences, Charité-Universitätsmedizin Berlin, Freie Universität Berlin, Humboldt-Universität zu Berlin, Berlin, Germany; 2 German Center for Mental Health, partner site Berlin/Potsdam, Berlin/Potsdam, Germany

**Keywords:** childhood emotional abuse, childhood maltreatment, childhood trauma questionnaire, heart rate variability, wrist-worn devices

## Abstract

**Background:**

Childhood emotional abuse (CEA) is a prevalent form of childhood maltreatment (CM) and has been consistently linked to psychological distress and sleep disturbances. However, objective sleep measures and heart rate variability often fail to support subjective complaints about sleep.

**Objective:**

The aim of this study was to investigate whether healthy women with a history of CEA show more sleep-related hyperarousal and other signs of disturbed sleep compared to women without CEA measured by simple at home devices and questionnaires.

**Participants and setting:**

We recruited 65 adult women (mean age 29 years) who reported a history of CEA on the Childhood Trauma Questionnaire (CTQ) and an age-, education- and body mass index matched control group consisting of 64 women without CEA.

**Methods:**

Participants completed validated questionnaires assessing depression, pre-sleep arousal, and subjective sleep quality. Objective data on sleep/wake states and nocturnal heart rate variability were collected for one night using a wrist-worn device.

**Results:**

The CEA group showed higher depressive symptoms, pre-sleep arousal, and subjective sleep disturbances, while selected objective sleep parameters and HRV did not differ. Within CEA, regression analyses suggested that childhood physical abuse largely accounted for the association between overall childhood maltreatment and insomnia severity, even after controlling analyses of residualization and partial correlations.

**Conclusions:**

These findings demonstrate that women with CEA exhibit more depressive symptoms, pre-sleep arousal and subjective sleep complaints that are not fully reflected in objective sleep parameters. The results are in line with previous studies showing discordant results between objective measures and subjective sleep quality in patients with a history of trauma. As physical abuse contributed unique variance to subjective insomnia burden, we temper claims of pure CEA specificity and frame findings in terms of a mixed maltreatment profile. To account for more subtle differences between the two groups, future studies should focus on stage-specific autonomic assessments.

## 1 Introduction

Childhood maltreatment (CM) is a strong risk factor for mental and somatic disorders (1) and encompasses sexual, physical, and emotional abuse as well as physical and emotional neglect (2). As a subtype, childhood emotional abuse (CEA) is defined as non-physical forms of rejection or hostile treatment by a caregiver, such as the repeated restriction of a child’s movements, belittling, blaming, threatening, intimidating, discriminating, or mocking (3).

CEA is considered a particularly important form of CM due to its high prevalence. It is often underreported and can co-occur with other forms of maltreatment, making it challenging to determine its prevalence. However, it is recognized as a highly harmful form of maltreatment with significant long-term mental health consequences. A nationally representative study in the United States found that roughly 5% of adults reported a history of CEA (4). In a systematic review and meta-analysis, the prevalence of CEA in North America was found to be 28.4% for girls and 13.8% for boys (2, 5). Previous studies show important sex differences: women experience higher rates of emotional abuse and neglect and often exhibit stronger internalizing sequelae (e.g., depression, anxiety) even at lower exposure levels (6). Neurodevelopmental research further suggests that CEA can produce sex-specific alterations in brain structure and function that confer differential vulnerability in girls and young women (7).

As a significant risk factor for long-term consequences on victims’ mental and physical health (8), CM is associated with increased risks of cardiovascular disease, diabetes and bronchial asthma (9, 10). Also, it is positively associated with disorders such as major depression, bipolar disorder, post-traumatic stress disorder (PTSD), and substance abuse disorders (11, 12).

CEA in particular is strongly linked to major depressive disorder and psychosis (8), as well as PTSD and anxiety disorder and can even surpass the link between these disorders and other forms of childhood abuse, namely sexual and physical (3, 13, 14). The distinct pathways linking CEA to health consequences later on are not well known, but the resulting hyperarousal, as indexed by cortical and autonomic arousal, and sleep disturbances might be an important aspect (15–18): CEA has been associated with increased pre-sleep arousal and other subjective sleep indicators (19).

However, while there is mounting evidence of subjective sleep complaints in people with a history of CM, few studies have examined the link between CEA and sleep disturbances using objective measures. Objective assessments using actigraphy have linked CM to reduced sleep efficiency and broader sleep health impairments in adulthood, such as irregular patterns and diminished satisfaction (17, 20). However, contrasting findings show that although CM was linked to subjectively impaired adult sleep, objective measures such as actigraphy and physiological metrics showed no significant correlations with CM exposure (21).

In insomnia research, this pattern (subjective sleep disturbance exceeding what is detectable with physiological metrics) is often described under the term of “sleep misperception” (22, 23). Contemporary models propose that such discrepancies may stem from microstructural arousal patterns (23–25) as well as cognitive-affective biases that heighten the salience and appraisal of nocturnal experiences (22). Importantly, these mechanisms can be seen as modifiable, with evidence that elements of Cognitive Behavioral Therapy can shape subjective sleep experience independent of macrostructural sleep physiology (26, 27).

A well-established marker to measure autonomic nervous system functioning and thus to estimate autonomic hyperarousal in sleep is heart rate variability (HRV). A reduced HRV is associated with heightened sympathetic activity and autonomic dysregulation, which are indicative of autonomic hyperarousal (28, 29). Importantly, a low HRV is a strong risk factor for myocardial infarction and mortality (30, 31).

CM has been consistently associated with reduced vagally mediated HRV, reflecting autonomic dysregulation (32), which in turn contributes to sleep disturbances characterized by diminished HRV and heightened nocturnal arousal (33–35). In adults with depression, CEA is associated with lower resting-state HRV (36, 37). However, findings regarding objective sleep parameters and autonomic function in individuals with a history of CEA remain inconsistent (16, 18, 21, 38). These inconsistencies may be further compounded by confounding factors such as sex and menopausal status, which independently influence autonomic regulation and sleep (39, 40).

Consumer wearable devices are increasingly recognized as valid tools for sleep monitoring, with modern multi-sensor devices demonstrating high sensitivity for sleep detection compared to polysomnography. Recent recommendations from the World Sleep Society and Sleep Research Society support their use in research settings. This approach aligns with the broader movement toward ecologically valid, accessible, and cost-effective sleep assessment methods that can be readily implemented in larger-scale studies and clinical screening applications (41).

Taken together, it is not well understood whether and how a history of CEA is associated with objective sleep and autonomic physiological parameters such as HRV in adults. There are few studies on sleep in adult individuals with CEA, but studies specifically examining hyperarousal during sleep in individuals with CEA and using simple home-based monitoring are still lacking. The aim of this study was to fill this gap and examine the presence of sleep-related hyperarousal, measured by HRV, in healthy women with a history of CEA but without current somatic or mental disorders. We collected objective and subjective measures of sleep and sleep quality in a naturalistic setting using a simple, low-cost, and non-invasive method. The primary hypothesis was that participants with CEA would exhibit significantly lower HRV (indicating higher physiological arousal) during sleep compared to those without CEA. Additionally, we investigated further differences in subjective and objective sleep measurements between the two groups.

## 2 Materials and methods

### 2.1 Participants

Based on the aforementioned influences on HRV and sleep, inclusion criteria for both groups required participants to be women, at least 18 years old and premenopausal. The decision to enroll only female participants was motivated by epidemiological, clinical, and neurobiological evidence indicating important sex differences (6). Methodologically, restricting the sample to premenopausal women reduces heterogeneity and the risk of confounding by sex-linked differences (42).

To be able to take part and fill out the questionnaires, participants also had to be fluent in the German language. For the CEA group, there were additional inclusion criteria: A history of childhood emotional abuse, defined by a score of 9 or higher for emotional abuse in the Childhood Trauma Questionnaire (CTQ) (43). The CTQ is a widely used and well-validated retrospective self-report instrument and the most commonly used instrument for assessing CM in research (44). It shows excellent psychometric properties, including high internal consistency, good test-retest reliability and convergent validity with structured clinical interviews (45, 46).

Apart from the subscale for emotional abuse, sexual and physical abuse CTQ subscales were recorded and scored but did not serve as inclusion/exclusion criteria. Subscales for emotional and physical neglect were not analysed.

Severity of abuse was rated and is shown in **Table 1**. The CTQ manual classifies subscale scores: minimal (emotional abuse < = 8, physical abuse < = 7, sexual abuse = 5), low to moderate (emotional abuse > 8 and < = 12, physical abuse > 7 and < = 9, sexual abuse > 5 and < = 7), moderate to severe (emotional abuse > 12 and < = 15, physical abuse > 9 and < = 12, sexual abuse > 7 and < = 12), and severe to extreme (emotional abuse > = 16, physical abuse > = 13, sexual abuse > = 13).

**Table 1:** Sample characteristics.

Variable	CEA N=65	nCEA N=64	t	df	p
Age (years) (Mean ± SD)	29.26 ± 8.05	29.56 ± 7.65	−0.22	127	0.83
BMI (kg/m²) (Mean ± SD) *	22.82 ± 2.93	23.07 ± 2.77	0.49	123	0.62
School education (years) (Mean ± SD) *	11.76 ± 0.69	11.84 ± 0.54	−0.75	123	0.46
CTQ emotional abuse (Mean ± SD); severity (N)	17.08 ± 4.69 (13, 8, 44)	5.75 ± 1.71 (0, 0, 0)	18.27	80.92	< 0.001
CTQ physical abuse (Mean ± SD); severity (N)	8.17 ± 3.92 (10, 10, 7)	5.00 ± 0.00 (0, 0, 0)	6.53	64	< 0.001
CTQ sexual abuse (Mean ± SD); severity (N)	5.92 ± 2.35 (7, 7, 13)	5.08 ± 0.32 (4, 0, 0)	2.88	66.47	0.01
BDI sum (Mean ± SD)	7.80 ± 7.77	1.77 ± 2.19	6.03	74.24	< 0.001

CEA, group with history of childhood emotional abuse; nCEA, group without history of childhood emotional abuse; SD, standard deviation; BMI, body mass index; CTQ, Childhood Trauma Questionnaire; BDI, Beck Depression Inventory-II; N, numbers; Severity, CTQ severity (N low, medium, severe).

*BMI: 3 missings in the CEA and 1 in the nCEA group. Years of school education: 3 missings in the CEA and 1 missing in the nCEA group.

Exclusion criteria included any history of mental disorders besides mild depressive episodes and specific phobias that had subsided at least 5 years prior to study participation and had not required professional treatment. Further exclusion criteria were any history of somatic illnesses that were diagnosed during the last 5 years, current pregnancy, a body mass index (BMI) below 19 or above 30 kg/m², intake of medication containing corticosteroids or any change in medication in the last 3 years.

The study included a total sample of 129 women, 65 with CEA and 64 without. The study was approved by the ethics committee of Charité, Universitätsmedizin Berlin and conducted according to the Declaration of Helsinki.

### 2.2 Procedures

Participants were recruited through public announcements online and at various institutions in Berlin, mainly serving purposes of higher education. Interested individuals filled out an anonymous online survey which consisted of demographic variables like sex, age, education level and three subscales of the German version of the CTQ (43). After that, individuals were contacted and screened via phone. Here, further criteria for inclusion and exclusion, especially history of somatic and mental disorder and medication as described above, were evaluated. This telephone screening was followed by a first in-person appointment. Here, participants undertook the Structured Clinical Interview for DSM-V Axis I and II (SCID-I and SCID-II) (47) and a pregnancy test. If there was a positive screening for Axis I or II disorders or a positive result on the pregnancy test, participants were excluded. Based on the criteria for inclusion described above, individuals were either excluded, included in the non-CEA (nCEA), or included in the CEA group. Both groups were matched for age, years of education and BMI.

Next, participants filled out several questionnaires: The Insomnia Severity Index (ISI) (48) a brief self-report questionnaire designed to assess the severity of insomnia symptoms and consisting of seven items that evaluate the nature, severity, and impact of insomnia, with scores ranging from 0 to 28. The Beck Depression Inventory-II (BDI-II) (49), a widely used self-report instrument for assessing the severity of depression, including 21 items, each scored on a scale from 0 to 3, with total scores ranging from 0 to 63. The German version of the Pre-sleep Arousal Scale (PSAS) (50, 51), a self-report measure designed to assess cognitive and somatic arousal experienced before sleep during the previous two weeks, consisting of 16 items divided into two subscales: cognitive arousal and somatic arousal. The evening protocol of the German Society for Sleep Medicine (52) for measuring subjective time spent in bed and wake time after sleep onset (WASO), number of interruptions during sleep as well as sleep onset latency (SOL, defined as the moment participants lie down to sleep to when they fall asleep) was filled out on the day before the measurement night, and its morning protocol was completed on the next day, following the measurement night.

Afterwards, for measuring HRV, WASO, SOL, total sleep time (TST), and number of interruptions of sleep during the night, participants were given a smartwatch (Polar Vantage M) to wear for 24 hours. HRV was analysed after connecting to a Polar software program and measured using photoplethysmography. Following a proprietary function of the Polar watch, average HRV during four hours of sleep was evaluated as “RMSSD”-HRV (“root Mean Square of Successive Differences”) in milliseconds, representing an established variant of HRV. Additional objective sleep parameters, such as SOL, WASO and TST were recorded by the watch using a combination of actigraphy and photoplethysmography. Taken together, participants reported subjective sleep measures for the same night that was assessed with the wearable device.

The Polar Vantage M watch, a wrist‐worn and widely used consumer sleep tracker, demonstrates high accuracy for heart rate and HRV (53, 54). Similar to other sleep-tracking devices, however, nocturnal wake periods are sometimes insufficiently detected, which might result in an overestimation of total sleep duration (53, 55).

Participants provided written informed consent and could withdraw from the study at any time. They received monetary compensation (€50) for their participation.

### 2.3 Statistical analysis

Data analyses were carried out using SPSS 27.0 statistical software (IBM Inc., Chicago, IL, USA).

The Kolmogorov-Smirnov test was performed to test for normality and if it was significant, data were log-transformed using a natural logarithm (ln). For measuring between-group differences of independent groups we used t-tests; p-values less than 0.05 were considered statistically significant.

Besides the objective and subjective sleep measures, we computed intra-individual difference scores for each participant by subtracting the objective sleep measure from the corresponding subjective report, as it is well established in insomnia research (56). Thus, for each person we derived discrepancy scores for SOL, TST and WASO. Positive values indicate that the participant reported greater subjective values than were recorded objectively.

A hierarchical linear regression analysis was conducted to examine the predictors of insomnia severity. To disentangle shared from unique effects of CTQ subscales, the CTQ physical abuse score was regressed on the emotional and sexual abuse subscales. The unstandardized residuals were then entered as the predictor of ISI in a linear regression within the CEA subgroup. In addition, computed partial correlations between physical abuse and ISI while controlling for emotional and sexual abuse were computed. Assumptions of linear regression were assessed prior to analysis. Multicollinearity was evaluated using variance inflation factors (VIF), with values below 10 considered acceptable. Homoscedasticity was assessed by visual inspection of residual plots (standardized residuals versus predicted values). Normality of residuals was tested using the Shapiro-Wilk test, as sample sizes were below or around 50.

## 3 Results

### 3.1 Sample characteristics

Demographic and clinical data are shown in **Table 1**. The groups did not differ significantly regarding age, BMI, and years of school education.

As predefined by inclusion criteria, there was a significant difference in the emotional abuse score of the CTQ: The CEA group showed higher mean values than the nCEA group, not only on the emotional abuse scale but also on the physical and sexual abuse scales. However, the mean scores for physical and sexual abuse were substantially lower than those observed for emotional abuse. BDI scores showed a significant difference between the two groups with higher but subclinical self-rated depressive symptoms in the CEA compared to the nCEA group. Note that these participants did not have a diagnosis of a current depressive episode, as current psychiatric diagnoses led to exclusion.

### 3.2 Subjective and objective sleep measurements and HRV

**Table 2** shows the results of subjective and objective sleep measurements in the two groups.

**Table 2:** Sleep and heart rate related measurements: The values for Discrepancy SOL, Discrepancy WASO and Discrepancy TST are misaligned and not aligned at the left side as the other values are.

Variable	CEA N=65	nCEA N=64	t	df	p	Cohen’s d
ISI sum (Mean ± SD)	8.42 ± 4.70	3.41 ± 3.14	7.13	111.79	<.001	1.27
PSAS sum (Mean ± SD) *	28.52 ± 8.59	20.75 ± 4.29	6.39	89.04	<.001	1.16
PSAS somatic (Mean ± SD) *	12.23 ± 3.67	9.61 ± 1.82	5.04	88.68	<.001	0.92
PSAS cognitive (Mean ± SD) *	16.29 ± 5.91	11.14 ± 2.84	6.21	87.15	<.001	1.13
TST (Mean ± SD) (min)*	439.88 ± 88.38	435.39 ± 76.61	0.30	118	0.77	0.05
SOL (Mean ± SD) (min)*	23.45 ± 39.16	18.83 ± 24.54	0.76	112	0.45	0.14
WASO (Mean ± SD) (min)*	27.63 ± 12.07	26.00 ± 13.49	0.68	113	0.50	0.13
Sleep interruptions (N)*	28.21 ± 10.84	25.90 ± 10.08	1.20	116	0.23	0.22
HRV sleep (Mean ± SD) (ms)*	60.00 ± 27.88	57.17 ± 25.07	0.58	114	0.57	0.11
Ln HRV sleep (Mean ± SD) *	4.00 ± 0.44	3.95 ± 0.45	0.56	114	0.58	0.10
Discrepancy SOL (Mean ± SD) (min)*	1.44 ± 29.33	0.30 ± 18.44	0.24	80.73	.814	0.05
Discrepancy WASO (Mean ± SD) (min)*	−16.08 ± 19.46	−18.62 ± 15.35	0.72	86.80	.471	0.15
Discrepancy TST (Mean ± SD) (min)*	−16.52 ± 71.38	−17.05 ± 61.47	0.04	104.94	.966	0.01

CEA, group with history of childhood emotional abuse; nCEA, group without history of childhood emotional abuse; SD, standard deviation; min, minutes; N, number; ms, milliseconds; ISI, Insomnia Severity Index; PSAS, Pre-sleep Arousal Scale; SOL, sleep onset latency; WASO, wake after sleep onset; TST, total sleep time; HRV, heart rate variability; Ln, natural logarithm.

*PSAS (same for all three components of the PSAS): 3 missings in the CEA group. TST: 6 missings in the CEA and 3 missings in the nCEA group. SOL: 10 missings in the CEA and 5 in the nCEA group. WASO: 11 missings in the CEA and 3 in the nCEA group. Sleep interruptions: 7 missings in the CEA and 4 in the nCEA group. HRV sleep: 8 missings in the CEA and 5 missings in the nCEA group (same for Ln HRV sleep). Discrepancy SOL: 15 missings in the CEA and 8 missings in the nCEA group. Discrepancy WASO: 18 missings in the CEA and 8 missings in the nCEA group. Discrepancy TST: 6 missings in the CEA group and 3 missings in the nCEA group.

Subjective measurements showed significant between group differences. Specifically, the CEA group reported significantly higher scores on the Insomnia Severity Index compared to the nCEA. Values of the ISI in the CEA group were above the cut-off for mild insomnia (57). Similarly, pre-sleep arousal as measured by the PSAS was significantly elevated in the CEA relative to the nCEA group, also reflecting a large effect. This also holds true for the two subcategories, somatic and cognitive arousal. In both, the mean values in the CEA group were above general clinical cut-offs for normal sleepers.

Contrasting to this, there was no difference in any of the objective measures collected by the wrist-worn Polar watch. No significant group differences were observed in the objective sleep parameters, including TST, SOL, WASO and number of sleep interruptions.

Mean HRV measurements during sleep as the primary outcome parameter also showed no difference between groups.

The discrepancy scores for SOL, TST and WASO showed no significant difference between the two groups.

### 3.3 Regression analyses

To further evaluate the association of subjective sleep complaints measured by the ISI with HRV and objective sleep measures, as well as the different domains on the CTQ (sexual, physical, and emotional abuse), we conducted an exploratory hierarchical multiple regression to predict ISI scores within the CEA subgroup. Emotional abuse was entered in the first block; in the second block we entered the remaining predictors. Results are presented in **Table 3**.

**Table 3:** Linear regression analysis – CEA group.

Independent variable	B	SE	β	t	p	VIF	CI (B)
CTQ emotional abuse	-0.23	0.14	-0.24	-1.58	0.12	1.53	-0.52/0.65
CTQ physical abuse	0.45	0.16	0.43	2.78	0.01	1.63	0.12/0.78
CTQ sexual abuse	-0.05	0.24	-0.03	-0.23	0.82	1.21	-0.54/0.43
BDI sum without sleep item	0.15	0.09	0.26	1.77	0.09	1.50	-0.02/0.33
BDI sleep item	0.88	0.84	0.14	1.04	0.31	1.21	-0.84/2.59
HRV in sleep (ms)	0.01	0.02	0.09	0.66	0.51	1.24	-0.03/0.06
TST (min)	-0.00	0.01	-0.01	0.04	0.97	3.45	-0.02/0.03
SOL (min)	0.04	0.02	0.36	2.33	0.03	1.61	0.01/0.07
WASO (min)	0.05	0.08	-0.141	-0.67	0.51	2.98	-0.21/0.10

Dependent variable: ISI sum score. R=0.70, R²: 0.50.

ISI, insomnia severity index; CTQ, childhood trauma questionnaire; emotional/physical/sexual, scores for emotional; physical and sexual abuse in the CTQ; BDI, Beck Depression Inventory-II; SOL, sleep onset latency; WASO, wake after sleep onset; TST, total sleep time; HRV, heart rate variability; ms, milliseconds; min, minutes.

The assumptions of linear regression were met. Multicollinearity was not a concern, as all variance inflation factors were below the critical threshold of 10, indicating acceptable independence among predictors. Visual inspection of residual plots revealed no systematic patterns such as funneling or wedge-shaped distributions, suggesting homoscedasticity. The Shapiro-Wilk test indicated that the standardized residuals did not significantly deviate from normality (p = 0.99). Childhood physical abuse was a significant independent predictor of insomnia severity in the CEA group. Amidst objective sleep parameters, SOL also made significant contributions to ISI.

In this subgroup, residualized physical abuse significantly predicted ISI (see **Table 4**), indicating that the component of physical abuse not shared with emotional or sexual abuse is associated with greater insomnia severity. The partial correlation between physical abuse and ISI (controlling for emotional and sexual abuse) corroborated the residualization result. CTQ subscales were moderately intercorrelated and VIFs were within acceptable limits, so while unique physical abuse variance predicted ISI, shared maltreatment variance remained substantial.

**Table 4:** Linear regression analysis on residuals (physical abuse)– CEA group.

Independent variable	B	SE	β	t	p	VIF
Unstandardized residual (physical abuse)	0.392	0.160	0.295	2.449	0.017	1.00

Dependent variable: ISI sum score. R=0.72, R²: 0.087.

To clarify whether the BDI sleep item alone accounted for the association with ISI, we separated the BDI into the sleep item and the remaining total score and entered both into the full model. This analysis showed that neither the isolated sleep item nor the remaining BDI score contributed independently to ISI.

Taken together, these results suggest that in the CEA subgroup, a history and the severity of childhood physical abuse and objectively measured SOL are significant independent predictors of insomnia severity, whereas the severity of CEA is not.

## 4 Discussion

The primary aim of this study was to investigate subjective and objective sleep parameters in healthy women with and without a history of CEA, including sleep-related hyperarousal, as indexed by nocturnal HRV and subjective pre-sleep arousal. We used simple, non-intrusive and low-cost methods to measure these parameters in a naturalistic home-based setting.

Participants with CEA reported significantly greater scores for subjective sleep complaints (ISI), Pre-sleep arousal (PSAS) and depressive symptoms (BDI). However, the objective sleep parameters (SOL, TST and WASO) and sleep-related HRV did not differ between the groups; also, intraindividual discrepancy scores for SOL, WASO, and TST showed no significant group differences. Further exploratory analyses suggested a prediction of a higher ISI in the CEA group by higher scores of childhood physical abuse and by SOL, but not by CEA. These findings invite several interpretations, as it suggests a pattern of subjective sleep complaints and worse subjective sleep quality not mirrored by nocturnal HRV and objective sleep parameters as measured in this study. Moreover, childhood physical abuse rather than emotional abuse appears to drive the association between childhood maltreatment history and elevated insomnia severity in this study.

Supporting the findings of our study, several lines of research indicate that a history of trauma is frequently reflected in elevated self-reported psychological symptoms around sleep, even when autonomic and objective sleep measures show no parallel disturbance. Evidence for subjective sleep complaints in people with a history of CM is vast: A meta-analysis from 2022 for example showed strong links between CM and symptoms of insomnia, adding to the literature with similar findings (58–60). A study examining overall CM and its different subtypes suggested that emotional abuse and neglect drive the association with subjectively impaired adult sleep. Objective actigraphy and physiological metrics, however, showed no significant correlations with CM exposure (21).

The observed non-accordance of subjectively low sleep quality and objective sleep measures is a well-established phenomenon in insomnia and trauma research. Here, the perception of being awake during certain sleep stages has been termed “sleep misperception” (22, 23). This mismatch is not unique to insomnia and is also observed in other clinical populations, such as women with CEA and physical comorbidities, as well as PTSD (61, 62).

There are several proposed mechanisms underlying this mismatch. Spectral analysis studies discuss differences in arousal-linked microstructure, whereas cognitive-affective biases around sleep are central to cognitive models of insomnia (23, 63–65). The observation that standard polysomnography often fails to detect substantial differences in sleep architecture led to the use of more nuanced neurophysiological methods, such as spectral EEG analysis and event-related potentials, which have revealed microstructural differences in REM and NREM sleep between insomnia patients and normal sleepers (63, 66). These microstructural changes, including increased high-frequency EEG activity during sleep, are associated with the subjective experience of wakefulness during sleep and may underlie sleep misperception (23–25). A recent study (67) emphasizes that misperception of sleep varies with microstructural markers (e.g., cortical arousals), but also with difficulties in emotional acceptance, supporting a model in which brief, sub-threshold arousals and lighter sleep stages amplify wake-like mentation and retrospective perceptions of poor continuity. In line with this, our null findings for standard objective markers do not rule out the presence of microstructural events or night-to-night instability that could shape subjective experience of poor sleep.

Cognitive models of insomnia locate the subjective–objective sleep mismatch primarily in heightened cognitive arousal, selective attention, and trait cognitive–affective biases, which amplify nocturnal imagery, worry, and memory for wake-like moments (22). They propose that pre-sleep worry and rumination increase the salience of internal sensations and external cues (e.g., the clock), producing sustained cognitive arousal that both delays sleep onset and biases retrospective estimates of sleep duration (68). Moreover, individuals who habitually monitor for sleep-related threat show greater selective attention to body sensations and environmental signs of not sleeping, which promotes overestimation of sleep-onset latency and underestimation of total sleep time (69). Imaging studies further indicate that subjective–objective discrepancy maps onto altered brain activity during sleep in networks implicated in salience and self-referential processing, suggesting that heightened cortical or network-level activation during NREM/REM may sustain conscious-like mentation and the feeling of wakefulness (26, 70). Thus, cognitive–affective biases and transient arousal events may interact: trait vulnerabilities increase the likelihood that brief micro-arousals or intrusive imagery will be noticed, encoded, and later recalled as evidence of poor sleep (27).

Although we did not observe an intra-individual mismatch between corresponding subjective and objective quantitative sleep measures, the poorer subjective sleep quality reported by the CEA group may indicate an elevated subjective burden: The pronounced elevations in depressive symptoms and pre-sleep arousal in this group could reflect heightened interoceptive attention and negatively biased cognitive processing, which may intensify the perceived severity of sleep disturbances. The elevated ISI scores may therefore reflect a tendency toward more negative appraisal of sleep or increased sleep-related worry, enhancing the salience and recall of poor sleep experiences without necessarily producing detectable differences in conventional objective indices.

Clinically, cognitive models imply that targeting attentional focus, maladaptive beliefs, and imagery processes is essential when subjective distress is high despite normal objective indices: interventions that reduce pre-sleep worry, interrupt monitoring behaviors, and modify trauma-related imagery should reduce subjective burden even if gross PSG metrics remain unchanged (26, 27);

In individuals with sleep misperception, Cognitive Behavioral Therapy for Insomnia (CBT-I) has been shown to reduce misperception and improve subjective sleep quality (71). The core components of CBT-I, sleep restriction, stimulus control, and cognitive restructuring consistently demonstrate meaningful therapeutic benefit (72, 73). In the specific population of this study (healthy women without clinical insomnia and a history of CM) however, treatment implications may need to extend beyond general CBT-I recommendations and interventions targeting perceptual and cognitive components of the sleep experience appear particularly relevant. Imagery-based approaches, known to be effective in nightmare disorders (74), offer a framework for addressing the subjective dimension of sleep disturbance. A RCT (75) demonstrated that imagery rescripting and imaginal exposure can reduce subjective sleep distress in nightmare disorder, even when physiological indices remain unchanged. Another study (76) highlights the utility of imagery rescripting for trauma-related sleep disturbances in PTSD, providing a clinically meaningful model for trauma-focused sleep intervention. More broadly, imagery-based self-regulation techniques (77) may help modify maladaptive cognitive-affective processes that shape subjective sleep perception.

As a marker for autonomic hyperarousal, we also compared nighttime HRV between the two groups. When including autonomic metrics like HRV, available literature is more heterogeneous. A history of CM could be linked to increased pre-sleep arousal on self-report scales, whereas baseline autonomic function (including resting daytime HRV) did not significantly differ from that of non-traumatized individuals (18). Evidence linking childhood maltreatment to reduced HRV is inconsistent: large-scale sleep data indicate lower RMSSD in those with CM (78), whereas daytime resting HRV shows no uniform effect across populations (79). Importantly, the CM–HRV relationship appears moderated by age and current psychopathology (55), and some studies and meta-analytic subsamples find effects only in clinically affected groups (80). It is important to note that most of these studies included people with heterogeneous forms of CM and sometimes current mental disorders, rather than specifically examining CEA in a healthy sample. Furthermore, most studies have assessed HRV during daytime resting states, whereas the present study uniquely targeted HRV during sleep.

This might explain why our findings concerning HRV in sleep in CEA differed from previous studies mentioned above: we examined healthy young women, mostly students or academics. As such, our sample might be a particularly resilient group. The fact that subjective sleep complaints were nevertheless associated with CTQ scores even in a resilient sample underscores the robustness of these perceptual and cognitive correlates of early adversity. This perspective also strengthens the use of intervention approaches that explicitly target cognitive-affective components of sleep experience, such as maladaptive sleep-related beliefs or attentional biases.

Notably, SOL emerged as an independent predictor of insomnia severity within the CEA subgroup in our analysis, even though there was not a significant difference between groups. This finding is consistent with research demonstrating that SOL is of particular clinical relevance, as it is the objective sleep parameter most closely linked to subjective sleep quality perception (81).

Taken together, our findings converge with the literature supporting a mismatch between subjective sleep complaints and objective findings, a pattern characteristic of insomnia and related conditions, even in the absence of macrostructural sleep abnormalities (22–24, 66).

Given that our study aimed to examine the physiological correlates of subjectively experienced CEA, the CTQ seems to be an appropriate and well-established assessment tool for participant classification. However, the literature consistently demonstrates that the types of maltreatment are highly interrelated, both in population-based and clinical samples (82–84). A latent profile analysis identified “high physical and emotional maltreatment” as a naturally occurring pattern, suggesting these abuse types frequently co-occur and may exert their effects through shared mechanisms such as impaired emotion regulation (85). This observation is also consistent with the high co-occurrence of maltreatment subtypes in our sample, which precluded the formation of a “pure” emotional abuse group. Indeed, we acknowledge the difficulty in differentiating the distinct groups of CM, especially during recruitment. We therefore treat our sample as characterized primarily by a history of childhood emotional abuse but acknowledge that other maltreatment types commonly co-occurred. The moderate intercorrelations among CTQ subscales imply a complex, mixed maltreatment profile rather than a pure CEA phenotype. Accordingly, we cannot claim CEA specificity and emphasize that our sample, which was recruited on the basis of emotional abuse scores, largely reflects overlapping maltreatment exposures.

Another finding of this study was that childhood physical abuse was the primary independent predictor of insomnia severity in the CEA group. This association may therefore index either a marker of greater overall maltreatment severity or a subtype-specific effect. This would align with a study that reported that both emotional and physical abuse were associated with heightened sleep reactivity, whereas sexual abuse was not (86). However, when controlling for multiple maltreatment types simultaneously, as in our regression analysis, physical abuse may emerge as the stronger predictor due to its potentially more direct physiological impact on stress regulatory systems. Due to the aforementioned rare distinction between different types of abuse in previous studies, further research is needed to clarify the observed effect of childhood physical abuse in this sample on subjective sleep quality (21).

### 4.1 Strengths & limitations

The present study offers several strengths alongside certain limitations: We employed naturalistic, ambulatory monitoring using a wrist-worn device in participants’ habitual sleep environments, thus enhancing ecological validity, and minimizing sleep laboratory-related artifacts. By combining self‐report scales with objective sleep metrics and nocturnal HRV, we capture a multidimensional picture of sleep and arousal. This is unlike most research, which in many cases relies on subjective measures or daytime resting‐state HRV.

Several limitations must be acknowledged. HRV and sleep parameters were assessed over a single night. This limits the reliability of sleep measures, as night-to-night variability is a recognized phenomenon in sleep research (87). HRV, the primary endpoint of this study, shows higher short-term stability across nights, suggesting that a single night may still provide reliable group-level estimates in healthy individuals (88).

The use of a wrist-worn device without polysomnography further precludes stage-specific autonomic profiling, which may reveal differences confined to particular sleep stages, such as REM (89). While the Polar Vantage M provides accurate heart rate and HRV estimates at rest (53, 54), its sleep tracking tends to overestimate total sleep time and underestimate wake periods (53, 55, 90), which may obscure subtle group differences. Wearable devices offer a practical and low-cost alternative to polysomnography, particularly for home-based monitoring, but PSG remains the gold standard for differential diagnosis and precise sleep staging (91, 92). Accordingly, null findings in objective measures should be interpreted with caution and may reflect the limits of the assessment strategy.

RMSSD was calculated to index high-frequency, vagally mediated HRV and is recognized as a cost-efficient time-domain metric for ambulatory settings (93, 94), but does not convey the balance of sympathetic and parasympathetic influences that the low frequency to high frequency (LF/HF) ratio aims to quantify (95). Relying solely on RMSSD might overlook sympathetic contributions and is generally less robust to artifacts than frequency-domain parameters (96). Accordingly, recent findings from our own group indicate that HRV alterations associated with CM may be more nuanced than previously reported (97). Another study found a trend towards lower resting-state high-frequency HRV in women with borderline personality disorder and high rates of CM (98). These observations underscore the value of assessing multiple autonomic dimensions and may help explain inconsistencies in studies relying primarily on vagal indices.

Another methodological limitation concerns the absence of menstrual cycle control. Both objective sleep measures and HRV show hormone-dependent fluctuations across the cycle (99): Polysomnographic studies report lower sleep efficiency during menses and the luteal phase compared with the mid-to-late follicular phase, prolonged SOL during menses, reduced TST, and increased nocturnal awakenings (99). HRV data indicate a reliable reduction in vagally mediated HRV from follicular to luteal phases (100). Rigorous phase determination requires multiple hormone assays, ovulation confirmation, and flexible scheduling, which was not feasible in this study (101). However, if participants were distributed across phases in both groups, this variability would likely affect both groups similarly.

Finally, participants were recruited based on elevated CTQ emotional abuse scores but were not excluded or stratified on other maltreatment subscales. Also, scores for physical and emotional neglect were not analysed. Several participants also reported physical and/or sexual abuse, limiting the ability to attribute effects uniquely to emotional abuse. As physical and emotional neglect were not assessed, some control participants may have experienced neglect despite reporting no abuse, which should be considered when interpreting between-group differences.

The study was conducted as an independent subproject within a larger research program with a predefined sample size and an *a priori* power analysis. With group sizes of 65 and 64, the design provided adequate power (0.80 at α = 0.05) to detect medium effects (Cohen’s d = 0.50), whereas smaller effects may not have been reliably detectable.

### 4.2 Conclusion

In summary, our study showed subjectively worse sleep quality in healthy women with CEA, that was not mirrored by objective sleep parameters measured with non-invasive wrist-worn devices.

Previously, very few studies examined sleep and measures of hyperarousal in this subgroup. Our observation shows similarities to the findings from studies in patients suffering from insomnia, suggesting the implementation of elements of CBT-I in treatment protocols in patients and corresponding preventive education in healthy individuals reporting CEA. However, differences in autonomic modulation may be more subtle and future studies should put a focus on stage-specific examinations as well as different parameters of HRV (inclusion of HF HRV and LF/HR ratio for example). To demonstrate a causal relationship of CM in adult hyperarousal and disrupted sleep, longitudinal studies are needed.

## Data Availability

The raw data supporting the conclusions of this article will be made available by the authors, without undue reservation.
